# Comparative swimming and station-holding ability of the threatened Rocky Mountain Sculpin (*Cottus* sp.) from four hydrologically distinct rivers

**DOI:** 10.1093/conphys/cox026

**Published:** 2017-05-04

**Authors:** Marie F. Veillard, Jonathan L.W. Ruppert, Keith Tierney, Douglas A. Watkinson, Mark Poesch

**Affiliations:** 1 Department of Renewable Resources, University of Alberta, 751 General Services Building, Edmonton, Alberta, Canada T6G 2H1; 2 Department of Biological Sciences, University of Alberta, G408 Biological Sciences Building, Edmonton, Alberta, Canada T6G 2E9; 3 Fisheries and Oceans Canada, Central and Arctic Region, 501 University Crescent, Winnipeg, Manitoba, Canada R3T 2NG

**Keywords:** Anaerobic metabolism, benthic fish, body morphology, flow modification, oxygen uptake

## Abstract

Hydrologic alterations, such as dams, culverts or diversions, can introduce new selection pressures on freshwater fishes, where they are required to adapt to novel environmental conditions. Our study investigated how species adapt to natural and altered stream flow, where we use the threatened Rocky Mountain Sculpin (*Cottus* sp.) as a model organism. We compared the swimming and station-holding performance of Rocky Mountain Sculpin from four different hydrologic regimes in Alberta and British Columbia, including the North Milk River, a system that experiences increased flows from a large-scale diversion. We measured the slip (*U*_slip_) and failure (*U*_burst_) velocities over three constant acceleration test trials. *U*_slip_ was defined as the point at which individuals required the addition of bursting or swimming to maintain position. *U*_burst_ was defined as the point at which individuals were unable to hold position in the swimming chamber through swimming, bursting or holding techniques without fully or partially resting on the electrified back plate. We found individuals from the Flathead River in British Columbia (with the highest natural flow) failed at significantly higher *U*_burst_ velocities than fish from the southern Albertan populations. However, there was no relationship between peak hydrologic flow from the natal river and *U*_burst_ or *U*_slip_. Further, *U*_burst_ velocities decreased from 51.8 cm s^−1^ (7.2 BL s^−1^) to 45.6 cm s^−1^ (6.3 BL s^−1^) by the third consecutive test suggesting the use of anaerobic metabolism. *U*_slip_ was not different between trials suggesting the use of aerobic metabolism in station-holding behaviours (*U*_slip_). Moreover, we found no significant differences in individuals from the altered North Milk River system. Finally, individual caudal morphological characteristics were related to both slip and failure velocities. Our study contributes to the conservation of Rocky Mountain Sculpin by providing the first documentation of swimming and station-holding abilities of this benthic fish.

## Introduction

Flow rate in lotic ecosystems defines basic environmental conditions from which organisms have evolved over the course of millions of years (Poff *et al*., 1997; Lytle and Poff, 2004). During the Anthropocene, close to half of the world's major river systems have been altered through the construction of dams and diversions for anthropogenic use (Lehner *et al*., 2011). For example, there are over 10 000 dams in Canada constructed for hydropower, irrigation, water supply, and mine tailings (Canadian Dam Association, 2003). These alterations of natural flow regimes can impact the amount and connectivity of suitable fish habitat, alter energy flow in the food web, increase the probability of the establishment of invasive species, disrupt cues for spawning and migration, impact riparian vegetation density and diversity, and affect the ability of some fish species to successfully complete all necessary life stages (Poff *et al*., 1997). Thus, altered flow regimes can influence the basic properties that support fisheries productivity, ecosystem health and clean water resources.

Hydrologic alterations have been attributed to the imperilment of approximately 39% of freshwater fish species in North America (Ricciardi and Rasmussen, 1999; Dudgeon *et al*., 2006; Jelks *et al*., 2008). This threat exemplifies the need to quantify impacts of flow modification on fish species. Swimming performance is a methodology that can be used to test species adaptations to flow through manipulated velocity experiments. Swimming performance reflects morphological (Hynes, 1970; Webster *et al*., 2011), physiological (Milligan, 1996; Reidy *et al*., 2000) and behavioural adaptations for maintaining life in moving water (Webb, 1989; Vogel, 1994; Carlson and Lauder, 2010). In lotic systems, pelagic and benthic fishes utilize different techniques to maintain position against a unidirectional current. Pelagic species swim to hold station (Webb *et al*., 1996) compared to benthic fishes, such as sculpins (Family: Cottidae), that interact with the substrate to maintain position (Webb, 1989; Tierney *et al*., 2011). Further, fishes have the capacity for some combination of aerobic (i.e. sustained swimming) and anaerobic (i.e. burst swimming) activity based on life history strategies (Hammer, 1995; Kieffer, 2000; Lucas *et al*., 2001).

These life history strategies can be useful in deciding the appropriate swimming performance test. Swimming performance in fish has been predominantly assessed using incremental velocity tests to define critical swimming speed (*U*_crit_) (Brett, 1964). These tests were developed for Salmonids (Family: Salmonidae) and have been used extensively by ecologists over the past 50 years (Beamish, 1978; Nelson *et al*., 2002). However, concerns over the inability of *U*_crit_ tests to differentiate between aerobic and anaerobic capacity led to the development of alternative tests, such as constant accelerations tests (CATs; *U*_burst_), to measure the anaerobic scope of individuals (Reidy *et al*., 2000, 1995; Nelson *et al*., 2002). This flexibility allows ecologists to utilize ecologically relevant methodologies based on the life history of their test subjects (Nelson *et al*., 2002). For example, endurance swimmers, such as salmonids, have a far greater aerobic scope than burst swimmers, such as sculpins (Lucas *et al*., 2001). While many factors influence aerobic scope, like individual fitness (e.g. body measurements) (Nelson, 1990; Kieffer *et al*., 1994), the inherent differences between fish families makes some tests more suitable than others. Sculpins have as little as 3–5% red ‘aerobic’ muscle mass compared to salmonids that can have up to 20% red muscle mass (Lucas *et al*., 2001). Therefore, the use of *U*_burst_ tests for sculpins can more accurately describe swimming abilities by focusing on the anaerobic scope of activity (Lucas *et al*., 2001).

**Figure 1:**
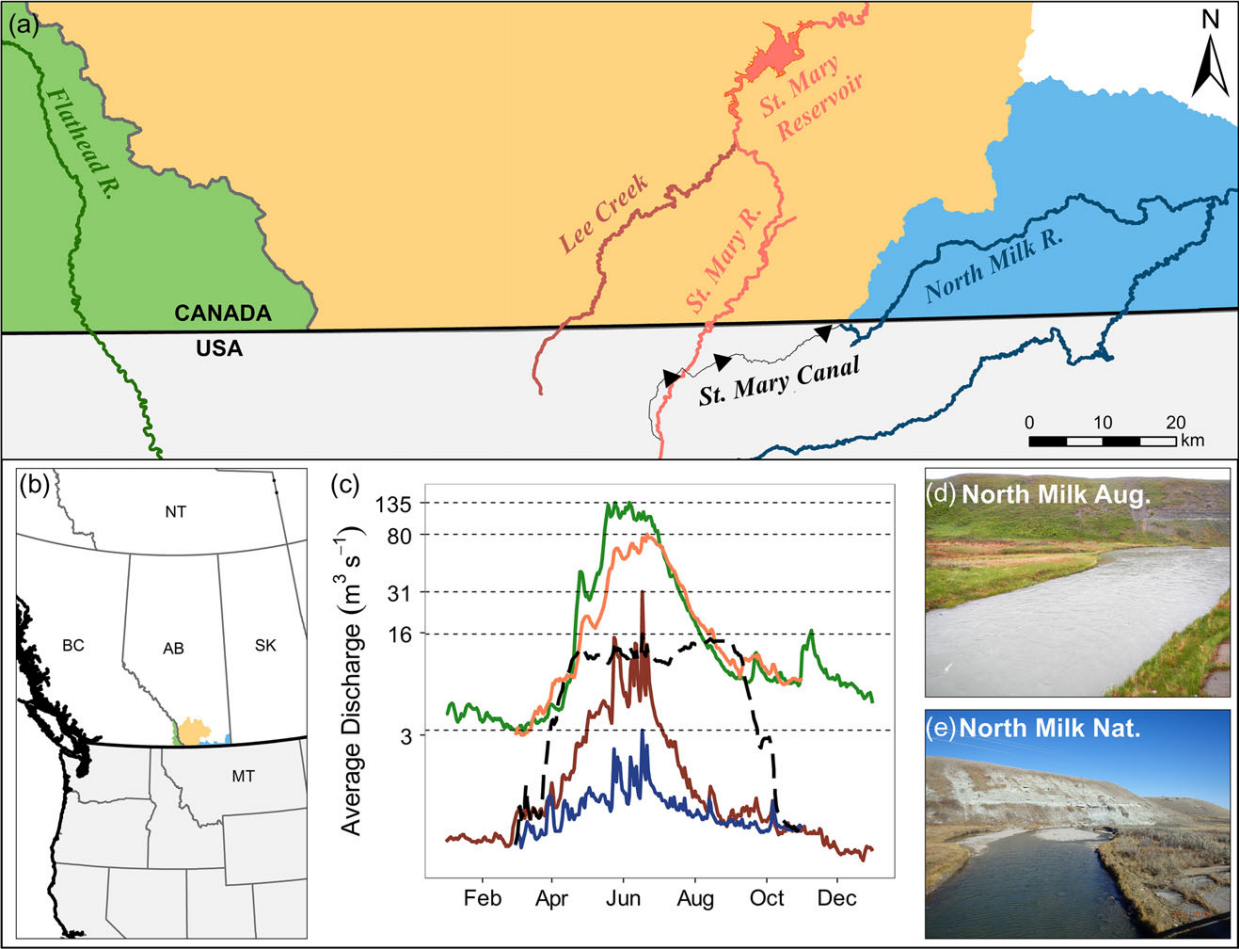
Canadian distribution of RMS in southern Alberta and southeast British Columbia (**a,b**). Polygons are coloured by subwatershed: Flathead River subwatershed (green), Oldman River subwatershed (orange) and Milk River subwatershed (blue). Within these subwatersheds, our study examined fish from the Flathead River (dark green), St. Mary River (orange), Lee Creek (dark orange) and North Milk River (dark blue). The North Milk River augmentation (St. Mary Canal) is shown in black. Inset (**c**) illustrates average discharge (m^3^ s^−1^) from 2008 to 2012 across the Canadian distribution of RMS taken from five hydrologic stations (Water Survey of Canada, 2015) plotted on a log10 scale. Peak discharge at each station is marked by horizontal dashed lines. Photos (**d**) and (**e**) show the same stretch of the North Milk River during augmented (aug.) and natural (nat.) flows.

Rocky Mountain Sculpin (*Cottus* sp.) are a model organism to understand the impact of flow alteration on sedentary species. Rocky Mountain Sculpin (herein referred to as RMS) is a threatened species with a restricted distribution in Canada and was recently identified as a unique taxon (COSEWIC, 2005). Within the range of RMS in Canada, waterway alterations for irrigation have drastically changed habitat and flow regimes with the construction of the St. Mary Canal in 1917 (COSEWIC, 2005). The St. Mary Canal annually diverts 178 × 10^6^ cubic meters of water from the St. Mary River to the North Milk River (Bradley and Smith, 1984) during the irrigation period from May through September. This diversion drastically increases peak flow rates in the North Milk River (3 m^3^ s^−1^ to 16 m^3^ s^−1^) and maintains an artificially stable discharge throughout the irrigation period (Fig. 1) (Water Survey of Canada, 2015).

We investigate the effect of large-scale flow alterations on RMS by comparing swimming and station-holding performance across four genetically distinct sub-populations throughout the Canadian distribution (Ruppert *et al*., 2017). Five-year average peak discharge (2008–2012) differs drastically between lotic ecosystems within the RMS range, including the North Milk River (3 m^3^ s^−1^ and 16 m^3^ s^−1^ for natural and augmented, respectively), Lee Creek (31 m^3^ s^−1^; St. Mary River tributary), St. Mary River (80 m^3^ s^−1^) and the Flathead River (135 m^3^ s^−1^) (Fig. 1) (Water Survey of Canada, 2015) providing a unique study system to test adaptations to natal flow regime. Specifically, in this study we determine (i) whether there are population differences in the maximum flow rate at which individuals can sustain position; (ii) if repeated trials influence swimming and station-holding performance; (iii) if phenotypic characteristics exist that may contribute to superior swimming and station-holding performance under modified flow rates; and (iv) the respiration rates under minimal flow (5 cm s^−1^). It is expected that predisposition to both natural and altered natal flow regimes would produce selection towards differences in swimming and station-holding performance of RMS from the four rivers.

## Methodology

### Fish

RMS were collected throughout their Canadian distribution in Lee Creek (24 July 2014; 30 Oct 2014), St. Mary River (24 July 2014; 30 Oct 2014), North Milk River (24 July 2014) and the Flathead River (26 Aug 2015) using a backpack electrofishing unit (Smith-Root, Vancouver, WA, USA). Average water temperatures in southern Alberta, taken from 0900-1330 around the date of initial collection, were 19 ± 2°C (18 and 21 July 2014), 16 ± 1°C (20 and 23 July 2014) and 18 ± 1°C (19 and 21 July 2014) for Lee Creek, St. Mary River and North Milk River, respectively. Average water temperatures in October for Lee Creek and St. Mary River were 8 ± 2°C (1 Oct 2014) and 9 ± 2°C (2–7 Oct 2014). Average water temperature in the Flathead River was 8.9 ± 2.6°C (26 Aug 2015). Following field collection, fish were held instream in a flow-through container overnight and fasted to reduce excretions during transport (Harmon, 2009). The following day, 50 L coolers were filled with water from the natal stream and fish were transferred from the flow-through container into the cooler with a dip net. Up to 100 individuals were transported per cooler (max. density = 2 ind.L^−1^). Aeration was provided to each cooler for the duration of the transport to the University of Alberta aquatics facility.

RMS were held in 120 L static-flow tanks with 40–100 individuals per tank. Fish were fed crushed nutrafin sinking pellets and dissolved invertebrate cubes, five days a week. Water temperature was held at 12 ± 1°C, using filtered, dechlorinated municipal water on a 0800:2000 light to dark schedule. Shelters were placed in tanks to reduce stress levels for RMS throughout their time in the aquatics facility. Fish were held for 2–6 months prior to testing. RMS from Lee Creek (*n* = 25), St. Mary River (*n* = 20), North Milk River (*n* = 25) and Flathead River (*n* = 26) were randomly selected from the holding tanks to undergo testing.

### Respirometry

About 24 h prior to swim tests, experimental fish were isolated from the feeding schedule to reduce the effects of digestion on metabolic rate (Jobling, 1981; Clarke and Johnston, 1999). Up to 12 h prior to testing, a sculpin was dip-netted from a holding tank at random, and transferred into a dark container with minimal human contact. The test fish was transported to the testing room and immediately relocated into one of two Brett-type respirometers (*v* = 10 L) containing freshly flushed air saturated water (12 ± 1°C) with a low velocity (5 cm s^−1^) unidirectional current. Fish moved out of the transport container into the respirometer on their own and rested in the low velocity current without the need to swim. Respirometers were sealed and oxygen levels (mg L^−1^) were continuously measured every second to the nearest 0.01 mg L^−1^ overnight (2000-0800) using a fibre optic oxygen probe calibrated weekly (Loligo Systems, Viborg, Denmark). Oxygen measurements were automatically recorded using the respirometry software AutoResp (Loligo Systems, Viborg, Denmark). To reduce stress from external movements and stimuli, respirometers were placed behind black curtains and monitored by video. Freshwater was continually flushed through the outer bath to reduce warming of the inner, sealed test water.

A bacterial oxygen consumption (mg L^−1^) trial was conducted following the completion of the fish trials by measuring oxygen consumption in the respirometer without a fish from 2000-0800 with a 5 cm s^−1^ unidirectional current. Oxygen consumption was calculated over a 4 h period (0200-0600) using data from 4 to 5 h following introduction into the respirometer to reduce the impact of transportation and handling on metabolic rate (Jobling, 1981; Tierney *et al*., 2011). Fish oxygen consumption (mg L^−1^; *t*_0200_–*t*_0600_) was corrected by subtracting bacterial oxygen consumption (mg L^−1^; *t*_0200_–*t*_0600_) to account for respiration from sources other than the test fish. The corrected oxygen uptake for fish was standardized by hour for body weight of individuals (mg L^−1^ g^−1^ h^−1^). Trials were removed where temperature increased more than 5°C overnight or if there were equipment issues.

### Swimming and station-holding performance

Swimming and station-holding performance was tested the following morning on the same fish used for respirometry the previous night. Fish were tested through a repeated measures design where each individual was observed in a series of three confined area constant acceleration tests (CATs) to quantify anaerobic burst swimming and exercise recovery potential (Reidy *et al*., 2000). Water velocity in the respirometer started at 5 cm s^−1^ and was programmed to increase by 2.5 cm s^−1^ every 10 s until fish reached fatigue. Two measures of swimming performance were recorded during each test: failure velocity and slip velocity. Failure velocity (*U*_burst_), a metric incorporating both swimming and station-holding abilities, was defined as the point at which RMS were unable to hold position in the swimming chamber through swimming, bursting or holding techniques without fully or partially resting on the electrified back plate. To ensure each fish reached a true failure velocity without resting, RMS were encouraged to move away from the back plate by applying short electrical pulses (0.25 ± 0.03 V) or, if necessary, a temporary reversal of flow direction. Fish were considered to have failed if they: (i) were responsive to the electrical pulse but did not remove themselves from the back grate after approximately 5 s of resting or (ii) returned to resting on the back grate after a temporary flow reversal. In the second case, the original failure velocity prior to flow reversal was recorded. Slip velocity (*U*_*slip*_), a measure of station-holding ability, was defined as the point at which fish were no longer able to hold station against the current without swimming or bursting (Webb *et al*., 1996). RMS utilize holding behaviour at low velocities where they exhibit virtually no body movement. When velocities increase, RMS must add bursting techniques to their holding behaviour or fully transition to swimming. The first point at which individuals slipped backward from a station-holding position or required the addition of bursting or swimming techniques to maintain position was considered the slip velocity. Due to technical failures, slip velocity was not measured on nine individuals, including: Lee Creek (*n* = 3), St. Mary River (*n* = 3) and North Milk River (*n* = 3). Swimming and station-holding performance was measured in cm s^−1^ and this metric was used for all analyses. A second metric, body lengths per second (BL s^−1^) was calculated for comparison with other papers.

Once fish failed, velocity was returned to 5 cm s^−1^ for a 30 min resting period. Each individual was tested in three CAT trials (CAT 1, CAT 2 and CAT 3) to investigate exercise recovery potential following fatigue. After all three tests were completed, sculpins were anaesthetized in tricaine methanesulfonate (TMS; MS-222; 0.2 g L^−1^) and body characteristics were recorded. Body characteristics were measured to the nearest 0.1 g (weight) and 0.1 mm (total length, body width, body height, caudal width, caudal height and caudal length) using a digital scale and digital calipers, respectively. Body characteristics were summarized for fish used in analysis once temperature mistrials were removed.

### Analysis

**Figure 2:**
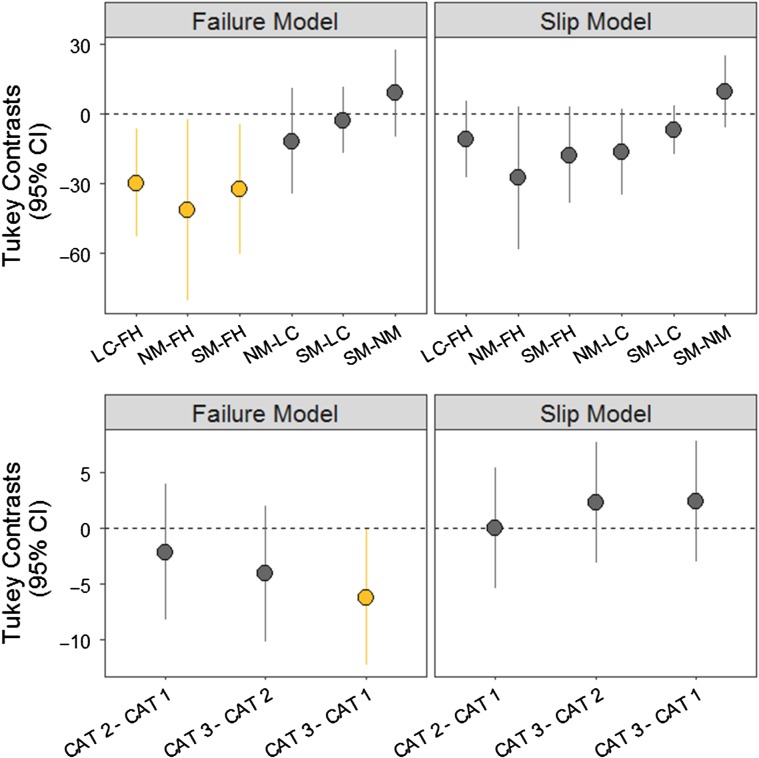
Tukey contrasts (estimate ± 95% confidence interval) between rivers (top row) and CAT trial numbers (bottom row) for failure (U_burst_) and slip (U_slip_) velocities from linear mixed-effects models; Significant differences (*P* < 0.05) are denoted in yellow; Rivers are abbreviated as follows: Flathead River (FH), St. Mary River (SM), Lee Creek (LC) and North Milk River (NM)

All analyses were conducted using the open sourced R statistical program (R Core Team, 2015). Factors influencing failure (*U*_burst_) and slip (*U*_slip_) velocities (cm s^−1^) were assessed using linear mixed-effects models in the *nlme* package (Pinheiro *et al*., 2014). Mixed-effects models are useful to deal with nested data, such as repeated tests on individuals, by allowing the intercept to vary for each individual (Zuur *et al*., 2009). Three groups of linear mixed-effects models were analysed for this study. Firstly, we analysed the effect of river and CAT trial number (fixed factors) on *U*_burst_ and *U*_slip_. These models were structured with each individual (FishID) as a random intercept to account for the repeated measures study design (Zuur *et al*., 2009). Covariates were included in each model to describe variation in swimming and station-holding performance due to the number of days held in the aquatics facility and the total length of each fish. This was necessary because of unevenly spaced holding times for each river and size differences between rivers. Attempts were made to standardize size of test individuals, however, one population (Flathead River) lacked sufficient fish within the targeted size. As covariates had difference scales and units, they were standardized and centred to a mean of zero in the R package *vegan* (Oksanen *et al*., 2016). Post-hoc Tukey tests were conducted to compare differences in *U*_burst_ or *U*_slip_ velocities between rivers and CAT trial number (Fig. 2) using the R package *multcomp* (Hothorn *et al*., 2008). Secondly, linear mixed-effects models were analysed to examine if there was a relationship between peak flow (m^3^ s^−1^) in natal streams and *U*_burst_ or *U*_slip_. These models were also structured with FishID as the random intercept. The model included covariates (days held and total length) and a fixed effect of peak flow (m^3^ s^−1^).

Finally, the impacts of body characteristics on *U*_burst_ or *U*_slip_ (cm s^−1^) were assessed using linear mixed-effects models with manual backwards selection (Zuur *et al*., 2009). These models were structured using a random intercept of FishID nested in River to account for repeated measures and river groupings from days held (as described above). This method allowed us to examine the effect of body characteristics (including total length) once the variation between rivers was removed. Fixed effects included: total length, caudal length, caudal height, caudal width, body height, and body width (Table 1). Fixed effects were centred as described above and collinearity was assessed using variance inflation factors in the R package *AED* (Zuur, 2010). VIFs were <10 indicating acceptable levels of collinearity.

**Table 1:** Summary of body characteristics and raw test results; Body characteristics were summarized for fish used in analysis; Failure (*U*_burst_) and slip (*U*_slip_) velocities are presented as both raw velocity (cm s^−1^) and body lengths per second (BL s^−1^); Results are presented as: mean (st. dev)

	Flathead River	St. Mary River	Lee Creek	North Milk River	All
Body characteristics	*n* = 26	*n* = 14	*n* = 17	*n* = 18	*n* = 75
Weight (g)	6.9 (3.8)	2.9 (1.7)	2.6 (0.7)	4.1 (1.5)	4.5 (3)
Total length (mm)	85.2 (12.4)	62.7 (8.4)	63 (4.6)	67.9 (6.9)	71.8 (13.5)
Caudal length (mm)	10.4 (2.2)	7.1 (1.6)	8 (1.1)	8.5 (1.3)	8.8 (2.1)
Caudal height (mm)	4.9 (0.9)	4 (1.3)	3.7 (0.4)	3.8 (0.6)	4.2 (1)
Caudal width (mm)	2.3 (0.4)	1.8 (0.6)	1.8 (0.6)	2 (0.3)	2 (0.6)
Body height (mm)	10.6 (2.4)	8.6 (2.1)	8.3 (1.4)	11 (1.8)	9.8 (2.3)
Body width (mm)	10.8 (2.5)	9.1 (1.9)	8.5 (1.4)	10.8 (1.7)	10 (2.2)
Failure velocity	*n* = 26	*n* = 14	*n* = 17	*n* = 18	*n* = 75
*U*_burst_					
CAT 1 (cm s^−1^)	68.8 (17.6)	44.4 (26.1)	39.7 (18)	44.4 (22.2)	51.8 (23.8)
(BL s^−1^)	8.3 (2.4)	7.1 (4.5)	6.3 (2.6)	6.6 (3.4)	7.2 (3.2)
CAT 2 (cm s^−1^)	66.3 (22.5)	39.1 (22.4)	41.4 (19.8)	41.7 (17.2)	49.7 (23.7)
(BL s^−1^)	7.9 (2.6)	6.4 (3.8)	6.6 (3)	6.1 (2.4)	6.9 (3)
CAT 3 (cm s^−1^)	59 (18.6)	37.5 (21.4)	36.7 (18.8)	41.1 (15.4)	45.6 (20.7)
(BL s^−1^)	7 (2.2)	5.9 (3)	5.8 (2.9)	6 (1.9)	6.3 (2.5)
Overall (cm s^−1^)	64.7 (19.9)	40.4 (23)	39.3 (18.6)	42.4 (18.2)	49 (22.8)
(BL s^−1^)	7.7 (2.5)	6.4 (3.7)	6.2 (2.8)	6.2 (2.6)	6.8 (2.9)
Test duration (min)	4.1 (1.3)	2.5 (1.5)	2.5 (1.2)	2.7 (1.2)	3.1 (1.5)
Slip velocity	*n* = 26	*n* = 11	*n* = 14	*n* = 15	*n* = 66
*U*_slip_					
CAT 1 (cm s^−1^)	21.2 (7.3)	24.3 (18.4)	27.3 (15.3)	19.1 (9.4)	22.5 (12.1)
(BL s^−1^)	2.5 (0.7)	4.1 (3.3)	4.3 (2.3)	2.9 (1.5)	3.2 (2)
CAT 2 (cm s^−1^)	22.9 (15.7)	19.7 (10.6)	21.8 (12.7)	25 (16.6)	22.6 (14.4)
(BL s^−1^)	2.7 (1.7)	3.4 (0)	3.4 (2)	3.8 (2.5)	3.2 (2)
CAT 3 (cm s^−1^)	24.9 (17.9)	21.1 (13.2)	23.7 (19.3)	29 (15)	24.9 (16.7)
(BL s^−1^)	3 (2.2)	3.6 (2.4)	3.8 (3.2)	4.4 (2.2)	3.6 (2.5)
Overall (cm s^−1^)	23 (14.3)	21.7 (14.1)	24.3 (15.8)	24.3 (14.3)	23.4 (14.5)
(BL s^−1^)	2.7 (1.7)	3.7 (2.5)	3.8 (2.5)	3.7 (2.2)	3.3 (2.2)
Oxygen measurements	*n* = 21	*n* = 13	*n* = 16	*n* = 16	*n* = 66
No detect (*n*)	16	7	10	0	33
Detect (*n*)	5	6	6	16	33
O_2_ Cons. (mg L^−1^ g^−1^ hr^−1^)	3.7 E-03 (3.7 E-03)	1.1 E-02 (9.4 E-03)	7.2 E-03 (7.2 E-03)	1.0 E-02 (9.0 E-03)	8.8 E-03 (8.3 E-03)

Backwards selection of fixed effects was performed using likelihood ratio tests to drop the least significant variables (Zuur *et al*., 2009). Corrected Akaike's information criterion (AICc_*i*_) for small sample sizes (Sugiura, 1978; Akaike, 1992; Bedrick and Tsai, 1994) was used to rank all final models created during backwards selection. Models were further compared using Δ_*i*_ (AICc_*i*_ –AICc_min_) and *w*_*i*_ (Akaike weights) to explain the strength of evidence for each model. Models with a Δ_*i*_ < 2 were considered similarly fitting models (Burnham and Anderson, 2004) and investigated further. To control for family-wise error due to multiple comparisons, *P* values for fixed effects were adjusted using the Holm–Bonferroni method (Rice, 1989).

## Results

### Swimming and station-holding performance between rivers and CAT trials

Overall, RMS swam to an average of 49 ± 22.8 cm s^−1^ (6.8 ± 2.9 BL s^−1^) over a duration of 3.1 ± 1.5 min before failing (*U*_burst_) (Table 1). RMS held station to an average of 23.4 ± 14.5 cm s^−1^ (3.3 ± 2.2 BL s^−1^) before slipping (*U*_slip_) (Table 1). The Flathead River fish swam to significantly higher *U*_burst_ velocities than those from the southern Albertan populations in Lee Creek (Tukey HSD, *z* value = −3.150, *P* < 0.001), St. Mary River (Tukey HSD, *z* value = −2.851, *P* = 0.01) and North Milk River (Tukey HSD, *z* value = −2.629, *P* = 0.03) (Fig. 2). There were no significant differences in *U*_slip_ between rivers (Tukey HSD, *z* value < 2.2, *P >* 0.09). Despite population differences in *U*_burst_, there was not a significant relationship between peak flow and *U*_burst_ velocities (df = 71, *t* value = 1.9, *P* > 0.05) or peak flow and *U*_slip_ velocities (df = 62, *t* value = 0.44, *P* = 0.66).

Significant differences in *U*_burst_ between repeated swim tests were observed from CAT 1 (51.8 cm s^−1^; 7.2 BL s^−1^) to CAT 3 (45.6 cm s^−1^; 6.3 BL s^−1^) (Tukey HSD, *z* value = −2.39, *P* = 0.0445) (Table 1, Fig. 2). However, *U*_slip_ showed no differences over CAT trials (Tukey HSD, *z* value < 1.042, *P* > 0.55) (Fig. 2).

### Body characteristics influencing swimming performance

Caudal width was an important variable influencing *U*_burst_ and *U*_slip_ and was included in all selected models (Table 2). Caudal width had a positive relationship with *U*_burst_ signifying fish with wider caudal measurements swam to higher velocities before failing (Table 3). However, Model 1 (*P* = 0.16), Model 2 (*P* = 0.07), and Model 3 (*P* = 0.27) showed caudal width was not significant (Table 3). Caudal width was additionally important for explaining *U*_slip_ and was included in both selected models (Table 2). Similar to failure velocity models, caudal width had a positive relationship with slip velocity but was not statistically significant in either Model 1 (*P* = 0.08) or Model 2 (*P* = 0.22) (Table 3).

**Table 2:** Linear mixed-effects models showing the effect of body characteristics on failure (*U*_burst_) and slip (*U*_slip_) velocity (cm s^−1^) using FishID nested in River as the random intercept. Model selection was calculated using the Akaike information criterion corrected for small sample size (AICc_*i*_); Models with a difference (Δ_*i*_) in AICc_*i*_ < 2 compared to the top model were considered to have substantial evidence and included in further analysis (shown in bold); Akaike weights (*w_i_*) further explain the strength of evidence for each model

	Models	Fixed effects	Random effect	Number of parameters (*K*)	AICc_*i*_	Δ_*i*_	*w*_*i*_
Failure velocity	**Model 1**	**Total length, caudal width, body width, caudal length**	**1| River/FishID**	**5**	**1969.61**	**0.00**	**0.41**
*U*_burst_ (*cms*^*−1*^)	**Model 2**	**Total length, caudal width, body width**	**1| River/FishID**	**4**	**1969.82**	**0.21**	**0.37**
	**Model 3**	**Total length, caudal width, body width, caudal length, body height**	**1| River/FishID**	**6**	**1971.37**	**1.76**	**0.17**
	Model 4	Total length, caudal width, body width, caudal length, body height, caudal height	1| River/FishID	7	1973.55	3.94	0.06
	Model 5	Total length, caudal width, body width, caudal length, body height, caudal height	–	6	1989.43	19.82	0.00
Slip velocity	**Model 1**	**Caudal height, caudal width**	**1| River/FishID**	**3**	**1622.91**	**0.00**	**0.57**
*U*_slip_ (*cm s*^*−1*^)	**Model 2**	**Caudal height, caudal width, body width**	**1| River/FishID**	**4**	**1624.87**	**1.96**	**0.21**
	Model 3	Caudal height, caudal width, body width, body height	1| River/FishID	5	1625.99	3.08	0.12
	Model 4	Caudal height, caudal width, body width, body height, total length	1| River/FishID	6	1627.89	4.98	0.05
	Model 5	Caudal height, caudal width, body width, body height, total length, caudal length	–	6	1628.75	5.84	0.03
	Model 6	Caudal height, caudal width, body width, body height, total length, caudal length	1| River/FishID	7	1629.92	7.01	0.02

**Table 3:** Parameter estimates from top linear mixed-effects models explaining failure (*U*_burst_) and slip (*U*_slip_) velocities as a function of body characteristics; Bolded text indicates variable significance at *α* = 0.05 after Holm adjustments

Response	Fixed effect	Coefficient	SE	*t* value	*P*
Failure velocity: *U*_burst_ (cm s^−1^)					
Model 1	Intercept	49.01	1.78	27.53	**<0.001**
	Total length	9.98	3.14	3.18	**0.01**
	Caudal width	4.34	2.20	1.97	0.16
	Body width	−5.45	2.82	−1.93	0.16
	Caudal length	3.41	2.28	1.49	0.16
Model 2	Intercept	48.94	1.91	25.66	**<0.001**
	Total length	11.84	2.91	4.07	**<0.001**
	Caudal width	4.77	2.20	2.17	0.07
	Body width	−5.63	2.89	−1.95	0.07
Model 3	Intercept	48.85	2.15	22.75	**<0.001**
	Total length	8.74	3.46	2.53	0.07
	Caudal width	4.13	2.21	1.87	0.27
	Body width	−7.03	4.35	−1.62	0.33
	Caudal length	3.42	2.30	1.49	0.33
	Body height	2.67	3.97	0.67	0.50
Slip velocity: *U*_slip_ (cm s^−1^)					
Model 1	Intercept	23.35	1.18	19.87	**<0.001**
	Caudal height	−2.10	1.51	−1.39	0.17
	Caudal width	3.14	1.51	2.08	0.08
Model 2	Intercept	23.35	1.18	19.75	**<0.001**
	Caudal height	−2.43	1.70	−1.43	0.32
	Caudal width	2.93	1.60	1.83	0.22
	Body width	0.70	1.65	0.43	0.67

Caudal length and caudal height were important for *U*_burst_ and *U*_slip_ velocities, respectively (Table 2), demonstrating the prominence of caudal morphology in swimming and station-holding performance. Caudal length had a positive relationship with *U*_burst_ but was not statistically significant in Model 1 (*P* = 0.16) or Model 3 (*P* = 0.33; Table 3). Alternatively, caudal height was negatively correlated with *U*_slip_, but was also not statistically significant in Model 1 (*P* = 0.17) or Model 2 (*P* = 0.32; Table 3).

Total length was included in all *U*_burst_ models, but was not included in the *U*_slip_ models (Table 2). Total length had a positive relationship with *U*_burst_ and was significant in Model 1 (*P* = 0.01) and Model 2 (*P* < 0.01) but not Model 3 (*P* = 0.07). Body width influenced both *U*_burst_ and *U*_slip_ velocities (Table 2). It was negatively correlated with *U*_burst_ (*P* > 0.07), and positively correlated with *U*_slip_ (*P* = 0.67) (Table 3). Body height was selected in one *U*_burst_ model and had a positive correlation (Tables 2 and 3). However, it was not significant (*P* = 0.5) (Table 3).

### Oxygen consumption

RMS oxygen consumption was detected in only half the trials (*n* = 33). All fish from the North Milk River (*n* = 16) had detectable oxygen uptake whereas only five fish were detected from the Flathead River and six were detected from each the St. Mary River and Lee Creek (Table 1). Due to small sample sizes and large variation between samples, we were unable to statistically compare differences between rivers.

## Discussion

RMS swimming and station-holding performance, as measured by failure velocity (*U*_burst_), was significantly different between individuals from the Flathead River and the southern Albertan populations. The Flathead River is separated from southern Alberta by the continental divide. This separation has resulted in strong genetic differences between populations from the Flathead River and populations from southern Alberta (Ruppert *et al*., 2017). Because we held fish in the aquatics facility for 2–6 months prior to testing, our fish were ‘detrained’ (Nelson *et al*., 2008) and measurements were a reflection of genetic differences in swimming and station-holding ability between populations (i.e. any phenotypic plasticity due to the flow regimes would not be expected to persist). The differences we found in swimming and station-holding between fish from either side of the continental divide are consistent with the strong genetic structuring of sub-populations. We did not, however, find any differences in fish from the augmented North Milk River compared to other populations despite strong population differences (Ruppert *et al*., 2017). RMS from the North Milk River have had one hundred years to adapt to seasonally augmented flows since the instalment of the St. Mary Canal in 1917 (COSEWIC, 2005). This corresponds to approximately 20 generations (COSEWIC, 2005) for genetic adaptations to arise. While genetic differences indicate the North Milk River may be a unique sub-population (Ruppert *et al*., 2017), in our study these genetic differences were not manifested in RMS swimming and station-holding ability from the North Milk River.

Contrary to our hypothesis, we did not see a gradient of swimming and station-holding performance correlated with the peak flow (m^3^ s^−1^) indicating that genetic differences in swimming and station-holding, as measured in this study, may not be strongly influenced by natal hydrologic regime. While phenotypic differences in RMS swimming performance may exist, a review of over 80 studies indicated the influence of flow on phenotypic plasticity in swimming performance is unclear (Langerhans, 2008). In the case of RMS, large-scale differences in hydrologic regime between systems may be diminished by strong microhabitat selection within these watersheds. For example, RMS utilize interstitial spaces created by physical substrate near the stream bed to complete their life history (Bailey, 1952; Finger, 1982; Haro and Brusven, 1994) by feeding on invertebrates and small fish on rock surfaces (Greenberg, 1991) and spawning under unembedded cobbles (Bateman and Li, 2001). These microhabitats can have velocities close to zero, called the boundary layer, as described by the Prandtl–vonKarman velocity equation (Chow, 1959; Hynes, 1970; Jowett, 1993). By carrying out their life history in the benthos, RMS can evade strong currents through the selection of unembedded cobble refugias (Bailey, 1952; Finger, 1982; Facey and Grossman, 1992). In a study on the energetic costs associated with microhabitat use in relation to velocity, Facey and Grossman (1992) found Mottled Sculpin (*Cottus bairdi*) selected microhabitat with velocities <1 BL s^−1^ despite their ability to hold station up to 5.8 BL s^−1^ (Facey and Grossman, 1992). As a result, microhabitat selection may shelter RMS from experiencing the full impact of broad-scale hydrologic regimes, thereby reducing the selection for swimming adaptations.

Averaged over all CAT trials, RMS swam to 6.8 ± 2.9 BL s^−1^ before failing (*U*_burst_). While no other studies have assessed the swimming and station-holding performance of RMS, our findings fall within the range of closely related cottids, such as Slimy Sculpin (*Cottus cognatus*) and Mottled Sculpin (*Cottus bairdi*) that can swim up to velocities of 9.4 BL s^−1^ (Webb 1978) and 5.8 BL s^−1^ (Facey and Grossman, 1990), respectively, in modified *U*_crit_ trials. Additionally, our study indicated that *U*_burst_ in subsequent CAT trials decreased significantly after a 30 min rest period between tests. Throughout the test duration, RMS predominantly held station up until the *U*_slip_ velocity, then transitioned into bursting-holding or bursting-coasting techniques until failure, as was noted in the round goby (Tierney *et al*., 2011). The significant decrease in failure velocity (*U*_burst_) over repeated CAT trials suggests the use of anaerobic metabolism in RMS consistent with patterns in repeat swim trials on other species. In a series of repeat CAT trials, European Sea Bass (*Dicentrarchus labrax*) exhibited the highest performance in their first CAT trial then subsequently declined (Marras *et al*., 2010). Similarly, repeated *U*_*crit*_ tests on Chinese Sturgeon (*Acipenser sinensis*) significantly declined over a series of four tests (Cai *et al*., 2014). Simultaneously, white ‘anaerobic’ muscle fibres contributed to swimming at lower velocities over repeated trials (Cai *et al*., 2014). During anaerobic metabolism, activity is largely fuelled by glycogen resulting in depleted energy stores and accumulation of waste products such as lactate (Milligan, 1996). Increased acidity from waste build up in tissues can impair oxygen delivery to muscles and inhibit aerobic respiration (Randall *et al*., 1987). Recovery periods, that can last up to 12 h in some species, replenish energy reserves and remove waste products (Milligan, 1996; Kieffer, 2000). Since our study allowed RMS to rest for only 30 min between *U*_*burst*_ tests, fish likely had lower energy reserves and higher waste accumulation as trials proceeded, hindering their ability to perform to the same level in subsequent trials. As bursting ability has been associated with predator evasion (Webb, 1986; Langerhans *et al*., 2004), reduced *U*_burst_ of RMS in subsequent tests suggests a hampered ability to repeatedly escape predators and other stressors. Alternatively, *U*_slip_ was not influenced by CAT trial number, indicating the use of aerobic respiration, which can sustain prolonged and repeated activity to the same level (Hammer, 1995). Marras *et al*. (2010) used the gait-transition speed (*U*_*gt*_), a similar metric to our *U*_slip_, to delineate the transition from aerobic to anaerobic metabolism. As seen in our study, *U*_*gt*_ was repeatable between trials and individuals (Marras *et al*., 2010). Consequently, the *U*_slip_ velocity may provide important conservation linkages to RMS microhabitat selection. Similarly to Mottled Sculpin (Facey and Grossman, 1992), RMS may select microhabitat with velocities far below their swimming performance abilities to remain within the scope of aerobic functioning and limit the need to switch to anaerobic respiration.

At an individual level, morphological characteristics helped explain intraspecific differences in swimming and station-holding performance. While previous studies have linked increases in body size to aerobic swimming performance (Hammer, 1995), burst anaerobic swimming can be propelled by caudal morphology in Gasterosteids (Webster *et al*., 2011) and Embiotocids (Drucker, 1996). Consistently, our study demonstrated the importance of caudal characteristics on both *U*_burst_ and *U*_slip_ metrics of RMS swimming and station-holding performance. Fish with wider and longer caudal peduncles were able to hold station against faster velocities resulting in both higher *U*_burst_ and *U*_slip_ velocities for these individuals. Moreover, caudal morphological characteristics can be shaped by flow velocities in Salmonids suggesting caudal morphology can respond to different hydrologic regimes (Imre *et al*., 2002; Peres-Neto and Magnan, 2004). Not only has the caudal region been implicated in burst swimming ability, Carlson and Lauder (2010) found that caudal morphology and position were important for station-holding postures in two species of darter (*Etheostomatinae*). Gait transitions leading into anaerobic burst swimming are often associated with the addition of caudal propulsion (Drucker, 1996; Svendsen *et al*., 2010; Webster *et al*., 2011) as the axial skeleton contains more muscle tissue than paired fins (Webb, 1998). In benthic fish, such as sculpins, anaerobic swimming is often preceded by station-holding, rather than steady aerobic swimming (Tierney *et al*., 2011) indicating a gait transition directly from holding to swimming. For these fish, morphological characteristics associated with anaerobic swimming are, therefore, central to the overall scope of swimming potential.

Finally, our study provided the first description of RMS resting metabolic rates. Although sample sizes were too small to compare significant differences between populations, fish from the North Milk River were detected more consistently than other populations despite the larger size of fish from the Flathead River. Due to the large-scale augmentation in the North Milk River, altered environmental conditions such as dissolved oxygen and temperature (Chabot *et al*., 2016) may impact the physiological fitness of RMS from this population. We suggest finer scale studies to assess the physiological cost of river augmentation on RMS using a smaller respirometer.

## Conclusion

This study is the first to describe the swimming ability of RMS, a newly identified and threatened benthic fish species in Canada. While we found differences in the *U*_burst_ between the southern Albertan populations and the Flathead River, broad-scale hydrologic regime did not influence the swimming or station-holding ability of this species. At an individual scale, morphological results indicated a selection of characteristics central to burst swimming. Further studies are required to determine the metabolic cost of flow augmentation on RMS for their long-term conservation.
